# Phasing of dragonfly wings can improve aerodynamic efficiency by removing swirl

**DOI:** 10.1098/rsif.2008.0124

**Published:** 2008-05-13

**Authors:** James R Usherwood, Fritz-Olaf Lehmann

**Affiliations:** 1 Structure and Motion Lab, The Royal Veterinary CollegeNorth Mymms, Hatfield, Herts AL9 7TA, UK; 2 BioFuture Research Group, University of UlmAlbert-Einstein-Allee 11, 89081 Ulm, Germany

**Keywords:** insect flight, biomimetics, wake capture, wing–wake interaction, particle image velocimetry

## Abstract

Dragonflies are dramatic, successful aerial predators, notable for their flight agility and endurance. Further, they are highly capable of low-speed, hovering and even backwards flight. While insects have repeatedly modified or reduced one pair of wings, or mechanically coupled their fore and hind wings, dragonflies and damselflies have maintained their distinctive, independently controllable, four-winged form for over 300 Myr. Despite efforts at understanding the implications of flapping flight with two pairs of wings, previous studies have generally painted a rather disappointing picture: interaction between fore and hind wings reduces the lift compared with two pairs of wings operating in isolation. Here, we demonstrate with a mechanical model dragonfly that, despite presenting no advantage in terms of lift, flying with two pairs of wings can be highly effective at improving aerodynamic efficiency. This is achieved by recovering energy from the wake wasted as swirl in a manner analogous to coaxial contra-rotating helicopter rotors. With the appropriate fore–hind wing phasing, aerodynamic power requirements can be reduced up to 22 per cent compared with a single pair of wings, indicating one advantage of four-winged flying that may apply to both dragonflies and, in the future, biomimetic micro air vehicles.

## 1. Introduction

Dragonflies are capable of a diversity of flight techniques, including effective gliding, powerful ascending flight, tandem flight during copulation, low-speed manoeuvring and hovering. In contrast to most other insects, direct musculature acts at each wing base, enabling dragonflies to control each wing independently. Indeed, a wide range of phase relationships have been described between fore and hind wings (Norberg 1975; Alexander 1984; Reavis & Luttges 1988; Wakeling & Ellington 1997*a*; Wang *et al*. 2003; Thomas *et al*. 2004). High flight forces have been correlated to in-phase flapping of fore and hind wings (Alexander 1984; Reavis & Luttges 1988; Rüppell 1989; Wakeling & Ellington 1997*a*), but in many instances dragonflies are also observed flying with fore and hind wings operating somewhat out of phase. Grodnitsky (Grodnitsky & Morozov 1993; Grodnitsky 1999) postulated that anti-phase wing motions might benefit hunting, either due to an increase in readiness for manoeuvrability, or by reducing centre of mass oscillations, both reducing visibility to potential prey and aiding location of targets. Previous computational (Wang & Russell 2007) and experimental (Luttges 1989; Maybury & Lehmann 2004) studies on the consequences of fore–hind wing phasing have demonstrated that phase can have a bearing on both thrust production and power. However, variations in thrust and power are closely correlated, suggesting that certain phases could increase thrust production, albeit with an increased power requirement, very much as would be expected with control through varying other kinematics such as frequency or angle of attack. Flow visualizations around flapping dragonfly models (Saharon & Luttges 1987, 1988, 1989) demonstrate the potential for interaction between the fore wing wakes and the hind wing, resulting in a range of possible consequences including the fusing of vortices and possible lift enhancement; however, their implications in terms of power and efficiency are not clear. An analytical study of heaving and pitching plates (Lan 1979), while not strictly directly applicable to hovering, and omitting the complications of separated flows subsequently believed to be a characteristic of much of slow dragonfly flight (Thomas *et al*. 2004), presents an exciting possibility: interacting tandem wings ‘can produce high thrust with high efficiency’ by ‘energy extraction by the hind wing from the wake of the forewing’. Further, visualization of smoke around free-flying dragonflies (Thomas *et al*. 2004) indicates the potential for a range of wing–wake interactions in forward flight. In this study, we use a mechanical model ‘hovering’ dragonfly to revisit the efficiency implications of phase on hovering with flapping, tandem wings.

## 2. Experimental details

**Figure 1:**
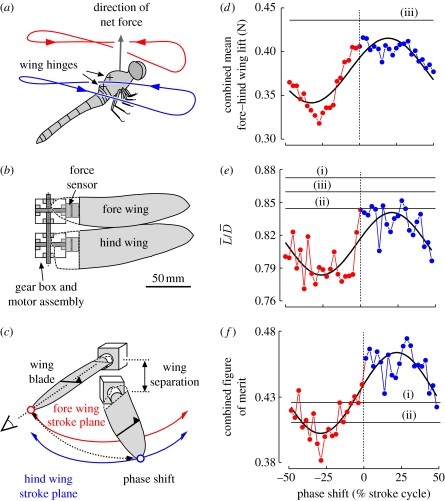
The mechanical dragonfly and results derived from force sensors at the wing bases. (*a*) The wingtip paths reported for a hovering dragonfly (Wakeling & Ellington 1997*a*) describe approximately horizontal stroke planes, with vertically stacked wings. (*b*,*c*) The mechanical model of a dragonfly's right wings was flapped at controlled fore–hind phases. Wing blade elements and gaze during flow visualization are indicated by the symbol and black lines plotted on the upper wing surfaces, respectively, in (*c*). The black triangle represents the wing's leading edge. Mean values derived from force sensors at the wing bases, of lift (*d*), the ratio of mean lift, $\bar{L}$, to mean drag, $\bar{D}$, (*e*), and aerodynamic efficiency expressed as ‘figures of merit’ (*f*) plotted as a function of fore–hind wing phase shift. Black solid lines show performances of isolated (i) fore wing, (ii) hind wing, (iii) cumulative effect of isolated fore and hind wings, and sine fit to combined-wing data as a function of phase.

We observed the effect of hovering with two pairs of wings by measuring the forces and wakes produced by an intermediate Reynolds number robotic hovering model dragonfly, and demonstrate the significance of hovering with a range of fore–hind wing phases. Reynolds numbers, based on the mean wing chord wing tip velocity, were 105 (fore wing) or 125 (hind wing); this is at the low end for small hovering dragonflies (calculated as between 250 and 500; Maybury & Lehmann (2004) from Rüppell (1989)), but is considered well above the transitional Reynolds number (Wang & Russell 2007). Fore–hind wing phases are described here as the proportion (per cent) of the stroke period at which the hind wing leads the fore: +25 per cent indicates that the hind wing leads the fore wing by a quarter cycle; 50 per cent indicates total anti-phase. The robotic model represents the two dynamically scaled right wings of a hovering dragonfly with realistic wing shapes and hinges vertically separated by 1.25 chord lengths, yielding fore wings beating directly above the hind wings (figure 1*a*–*c*). Both fore and hind wings followed identical, generalized kinematics sweeping a horizontal stroke plane (see the electronic supplementary material). The aim of the robot kinematics was not to precisely reproduce any single set of measured wing motions, but to provide a moderately realistic test bed with which the significance of wing phasing could be investigated without introducing confounding aerodynamic factors such as the direction of the net force vector. Thus, while a range of stroke plane angles have been described for hovering dragonflies, we selected vertically stacked horizontal stroke planes following the observations of hovering *Sympetrum sanguineum* (Wakeling & Ellington 1997*a*). Instantaneous aerodynamic lift, defined as the vertical force that provides weight support, and drag, the force impeding the motion of the wing in the horizontal plane, were measured with force sensors at the wing bases. From these values, we calculated the mean lift force, the ratio of mean lift to mean drag, the power required to overcome drag, and the aerodynamic efficiency. Aerodynamic efficiency is represented by the ‘figure of merit’ (FoM), a special case of ‘propeller efficiency’ used for hovering helicopters (electronic supplementary material). This term describes the ratio of the minimum theoretical power required for hovering to the measured aerodynamic power (Prouty 2005). In effect, the FoM expresses aerodynamic efficiency by comparison with an ideal helicopter.

**Figure 2:**
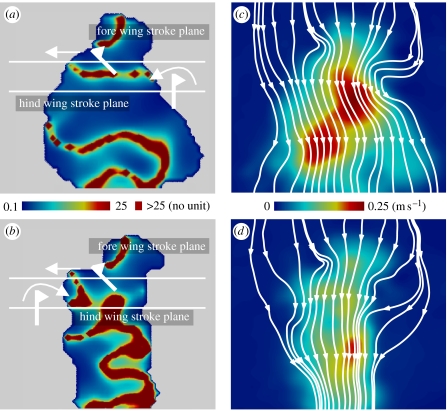
Wake patterns derived from two-dimensional digital particle image velocimetry at the instant of mid-downstroke of the fore wing, when the fore wing is directed directly towards the viewer, for (*a*,*c*) least efficient (−25%) and (*b*,*d*) most efficient (+25%) kinematic phase shifts. The wake of the efficient phase displays a higher ratio of downward to lateral velocities (*b* versus *a*, where the ratio is represented by colour). Flow regions below a vertical flow velocity threshold of 0.1 m s^−1^ are shown in grey. Streamtubes showing wake contraction in (*d*) compared with wake expansion in (*c*) indicate that less momentum, and less kinetic energy, is wasted as swirl at positive (hind wing leads) kinematic phase shifts. Fluid velocity for both flapping conditions is indicated by colour background in (*c*) and (*d*).

The fluid (mineral oil) inside a 0.43 m^3^ flow tank was seeded with air bubbles to allow visualization of a two-dimensional slice of the flow field around the wings and including the wake beneath the hovering mechanical dragonfly. The properties of these flows were quantified using digital particle image velocimetry (2D-DPIV, TSI Insight 6.0), and are presented for a vertical slice situated half way along the fore wing at the instant of mid-downstroke of the fore wing (figure 2). Video (electronic supplementary material) illustrates the effect of fore–hind wing phasing on the dynamic wake structure through several flapping cycles.

## 3. Results and discussion

Interaction between fore and hind wings was largely detrimental in terms of lift (figure 1*d*; Maybury & Lehmann 2004), agreeing with computational analysis (Sun & Lan 2004), though some phases are less detrimental than others (see also Wang & Russell 2007). The reduction in lift is attributable to a reduction in the angle of incidence between each wing and the local fluid: the wings produce an induced downward flow both below (downwash) and above (inwash) the level of the wings; angles of incidence are reduced due to the fore wing's downwash on the hind wing, and the hind wing's inwash on the fore wing. In addition to the reduced lift with fore–hind wing interaction, the mean lift to mean drag ratio of the flapping wings, while varying with phase, is not improved compared with wings operating in isolation (figure 1*e*). It is thus tempting to conclude that aerodynamic efficiency would always be reduced when wings operate in tandem. However, we find that this is not the case: at advanced phases, FoMs are better than in isolated wings.

Figure 1*f* shows that aerodynamic efficiency of isolated wings is poor in all cases, consistently below half that of an ideal actuator disc or perfect helicopter, approximately half of that calculated analytically without flow separation (Lan 1979), and considerably below values achievable by real helicopters (approx. 0.75; Prouty 2005). This is consistent with the very high angles of attack, flow separation and loss of leading-edge suction at the leading edges, and subsequent poor lift–drag ratios of hovering insect wings. However, the FoMs of isolated fore or hind wings are surpassed by combined fore and hind wings operating with positive wing phases: four-winged flight with correct fore–hind phasing improves aerodynamic efficiency. To determine aerodynamic power savings, we calculated the wing beat frequency required to achieve identical mean lifts (0.404 N) for wings operating at different phases. From this, the power requirements for hovering were scaled and compared: hovering with a phase shift of +25% requires 16 per cent less power than with a phase shift of −25%. Although this comparison ignores other potential kinematic parameters, it shows that hovering with the correct phase between fore and hind wings can have a considerable energetic significance. Similarly, a comparison can be made between two-winged and four-winged power requirements. Constraining wing shape and all aspects of kinematics apart from wing beat frequency, hovering with four wings at best phase shift requires 22 per cent less power than hovering with only the fore wings at the same mean lift production.

The apparent paradox that efficiency can be improved despite a reduced ratio of mean lift to mean drag is explained by two different views describing the same physical phenomenon. The first is a shift in the timing of the forces. The periodic, non-vertical components of the wake left by the fore wing allow, at positive, hind wing leading phase shifts, the hind wing to generate high aerodynamic forces when moving relatively slowly, at the extremes of the stroke. When operating at a phase shift of +25%, the hind wing experiences a peak in lift enhancement (lift compared with the hind wing flapping in isolation) at 35 and 85 per cent of the stroke period, towards the end of down and upstroke (Maybury & Lehmann 2004), respectively—when the wing is moving at approximately 80 per cent of its peak speed. As power is the vector product of drag and wing velocity, a bias of force development away from periods when the wing is moving fastest reduces the power requirements. Thus, while the significance of unsteady aerodynamic force development—that which cannot be predicted with a quasi-steady analysis relating aerodynamic forces to the square of velocity—within the stroke cycle is minor in terms of the total force production, it is considerable in terms of power and efficiency.

The second description of this phenomenon is apparent from the resultant wake after the action of both sets of wings (figure 2 and electronic supplementary material). The term ‘swirl’ applies to lateral motions of the wake representing non-downward, and thus non-weight-supporting momentum. Energy put into swirl is wasteful, as it is not associated with momentum flux providing weight support. At positive kinematic phases and high aerodynamic efficiencies, swirl applied to the fluid by the fore wings can be recovered to a certain extent by the hind wings, redirecting lateral motions of the wake into the vertical. This effect is illustrated with snapshots of the flow field showing that, at an inefficient (−25%) phase shift, the spreading wake has a considerable component of non-downward momentum (figure 2*a*). By contrast, at efficient (+25%) fore–hind phase shifts, the contracting wake is largely vertical (figure 2*b*). Streamtubes of the wake at the same instant and position highlight the effectiveness of the +25% phase in producing a conventional, converging momentum jet (figure 2*c*,*d*). Flow speeds in the wake are also generally lower at +25% phase shift, suggesting that less kinetic energy is put into the wake for a given change in vertical momentum, although a full three-dimensional flow field would need to be measured in order to quantify this phenomenon with PIV. The power required for a given mean lift force is reduced at a phase of +25% by a form of interwing wake recapture; this broadly matches the 28 per cent phase observed by Wakeling & Ellington (1997*a*) for a near-hovering (advance ratio of 0.21) free-flying dragonfly. The mechanism for improved efficiency by swirl removal matches the ‘energy extraction by the hind wing from the wake of the forewing’ predicted by Lan (1979), and is directly analogous to that exploited by coaxial contra-rotating rotors, exemplified by helicopters such as the Kamov Ka-50.

## 4. Conclusions

The finding that the conditions for high lift are the same as those for high aerodynamic efficiency raises the question of why dragonflies use such a diversity of kinematic phase shifts during free flight. Previous suggestions include varying requirements for thrust, efficiency and readiness for manoeuvrability and some other aspect of hunting performance (Grodnitsky 1999). An alternative explanation for the observed phase shifts in free flight is that the appropriate wing phasing to make effective use of swirl removal is dependent on the speed with which the wake travels between fore and hind wings, and this is determined by the flight speed, direction and thrust production (Wakeling & Ellington 1997*b*). In this scenario, the +25% phase shift between both wings during hovering should decrease with increasing flight speed owing to the increase in wake velocity relative to the dragonfly. The range of observed phase shifts might therefore simply reflect the kinematic requirements to achieve the same swirl-removing mechanism at the various flight conditions by ensuring that the hind wing meets the correct part of the fore wing wake. At this stage, the best direct evidence that such wing–wake interactions occur in free-flying dragonflies is the unstructured wake, ‘devoid of vortex loops’, and absent of starting vortices, described from smoke visualization (Thomas *et al*. 2004). This is consistent with the swirl-cancelling characteristic of the efficient phase relationship, which results in a predominantly downward wake (movie S2), and contrasts with the inefficient wake (movie S1), in which the stop/start vortices are maintained.

Caution must be applied when interpreting the biological significance of the above observations. Suggesting an evolutionary advantage to either two-winged or four-winged forms is unwise, considering the success and diversity of the true flies (Diptera), and yet the maintenance of the four-winged form by dragonflies since the Carboniferous. However, in terms of engineering, the findings presented here may be particularly valuable. Any energetic benefit from four-winged flapping would be of great interest in the field of biomimetic aircraft design (Stafford 2007) because flapping-winged aircraft are challenged by the high power requirements of flapping flight (Ellington 1999). Appropriately phased four-winged flapping, analogous to dragonfly flight, may thus present one aerodynamic trick to reduce these power requirements and improve the endurance of the next generation of flapping micro air vehicles.

## Supplementary Material

Additional material Kinematics and morphology; derivation of Figure of Merit; qualitative demonstration of the effect of phase on swirl

Additional figure

Illustrative film Film showing wake for innefficient phase

Illustrative film Film showing wake for efficient phase
