# Supplementary material for: Phasing of dragonfly wings can improve aerodynamic efficiency by removing swirl
Source: J R Soc Interface. 2008 May 13;5(28):1303–7. doi: 10.1098/rsif.2008.0124 (PMC2607445; doi:10.1098/rsif.2008.0124)
Supplement: Additional figure [file rsif20080124s02.doc]

**ELECTRONIC SUPPPLEMENTARY MATERIAL**

*Kinematics and morphology*

The wings were scaled, immersed in mineral oil, and operated in a 0.6 x 0.6 x 1.2 m^3^ glass tank at a flapping frequency of 0.533 Hz, following the kinematics shown in figure ESM1. Reynolds number for wing motion was 105 for the forewing and 125 for the hind wing. This is appropriate for the smallest dragonflies (Maybury and Lehmann 2004), experimentally convenient for flow visualisation using Particle Image Velocimetry, and the precise Reynolds number is unlikely to have a large bearing on force generation (Sane 2003). The wing planforms were taken from *Polycanthagyna melanictera*, a small Japanese dragonfly, because they have been well described in previous morphological and kinematic work (Wang *et al*. 2003), and are sufficiently similar to *Sympetrum*. The findings of this study are insensitive to precise wing shape; similar results are observed when fore and hind wings are replaced by a generic *Drosophila* planform.

*Derivation of Figure of Merit*

The ‘Figure of Merit’ FoM is a special case of propeller efficiency that may equally be applied to hovering helicopters and insects:

$FoM\text{ =}\frac{\text{Ideal} \text{induced} \text{power}}{\text{Total} \text{aerodynamic} \text{power}}$.

The total aerodynamic power was derived from the mean of the instantaneous powers (drag force multiplied by wing velocity) measured directly at the wing bases using a six degree-of-freedom force transducer (ATI nano17). The rate of useful work is the ideal induced power, or the minimum rate with which energy has to be added to the wake to provide the observed mean lift $\overline{L}$. This ideal Rankine-Froude induced power for hovering $\overline{P_{ind}}$ is given by:

,

with $\rho$ the fluid density and *A*_0_ the actuator disc area, the area swept by the wings. With vertically stacked wings operating with horizontal stroke planes, as for coaxial rotors, the flapping fore and hind wings form a single actuator disc – both sets of wings accelerate the same fluid. Following Ellington (1984), and considering the right wings of the dragonfly model, the appropriate total actuator disc area for both wings is:

, (3)

where Φ is the downstroke amplitude and *R* the wing length of the longer wing (forewing, 0.19 m).

## Qualitative demonstration of the effect of phase on swirl

The straightening of the wake – the mechanism by which efficiency is improved with the correct fore-hind wing phase relationship – can be observed qualitatively using almost neutrally buoyant air bubbles (raising speed <0.5 mm s^-1^) in the fluid. Movie S1 shows the worst case, with the forewing leading the hind wing by 25% of the stroke cycle. A considerable side-to-side motion is visible in the wake, showing momentum and energy wasted as swirl. By contrast, Movie S2 shows the best case phase, with the hind wing leading by 25%; the wake is predominantly downward.

**ESM additional references**

Ellington, C.P. (1984). The aerodynamics of hovering insect flight. V. A vortex theory. *Phil. Trans. R. Soc. Lond*. B **305**, 115–144.

Sane, S.P. (2003). The aerodynamics of insect flight. *J. Exp. Biol.* **206**, 4191-4208.

**Figure ESM1 legend**

The fore and hind wings followed identical, idealised dragonfly kinematics, where AoA denotes the angle of attack, and *U*_T_ the wingtip velocity.
